# A radiomics nomogram based on contrast-enhanced CT for preoperative prediction of Lymphovascular invasion in esophageal squamous cell carcinoma

**DOI:** 10.3389/fonc.2023.1208756

**Published:** 2023-07-03

**Authors:** Yating Wang, Genji Bai, Wei Huang, Hui Zhang, Wei Chen

**Affiliations:** Department of Radiology, The Affiliated Huaian No.1 People’s Hospital of Nanjing Medical University, Huaian, Jiangsu, China

**Keywords:** computed tomography, decision curve analysis, esophageal squamous cell carcinoma, lymphovascular invasion, nomogram

## Abstract

**Background and purpose:**

To develop a radiomics nomogram based on contrast-enhanced computed tomography (CECT) for preoperative prediction of lymphovascular invasion (LVI) status of esophageal squamous cell carcinoma (ESCC).

**Materials and methods:**

The clinical and imaging data of 258 patients with ESCC who underwent surgical resection and were confirmed by pathology from June 2017 to December 2021 were retrospectively analyzed.The clinical imaging features and radiomic features were extracted from arterial-phase CECT. The least absolute shrinkage and selection operator (LASSO) regression model was used for radiomics feature selection and signature construction. Multivariate logistic regression analysis was used to develop a radiomics nomogram prediction model. The receiver operating characteristic (ROC) curve and decision curve analysis (DCA) were used to evaluate the performance and clinical effectiveness of the model in preoperative prediction of LVI status.

**Results:**

We constructed a radiomics signature based on eight radiomics features after dimensionality reduction. In the training cohort, the area under the curve (AUC) of radiomics signature was 0.805 (95% CI: 0.740-0.860), and in the validation cohort it was 0.836 (95% CI: 0.735-0.911). There were four predictive factors that made up the individualized nomogram prediction model: radiomic signatures, TNRs, tumor lengths, and tumor thicknesses.The accuracy of the nomogram for LVI prediction in the training and validation cohorts was 0.790 and 0.768, respectively, the specificity was 0.800 and 0.618, and the sensitivity was 0.786 and 0.917, respectively. The Delong test results showed that the AUC value of the nomogram model was significantly higher than that of the clinical model and radiomics model in the training and validation cohort(P<0.05). DCA results showed that the radiomics nomogram model had higher overall benefits than the clinical model and the radiomics model.

**Conclusions:**

This study proposes a radiomics nomogram based on CECT radiomics signature and clinical image features, which is helpful for preoperative individualized prediction of LVI status in ESCC.

## 1 Introduction

Esophageal cancer is one of the most common malignant tumors, with high morbidity and mortality rate (1). The most common types of esophageal cancer are squamous cell carcinoma (ESCC) and adenocarcinoma (EA), and ESCC is the most common pathological type of esophageal cancer in China. The overall survival (OS) of patients with esophageal cancer remains poor despite significant improvements in diagnosis and treatment in recent years, with a 5-year survival rate of 15%-20% (2). Although great progress has been made in chemoradiotherapy for ESCC in recent years, esophagectomy is still the most effective treatment. However, more than half of patients undergoing radical esophagectomy develop local recurrence or distant metastasis within three years (3).In order to develop individualized treatments for ESCC, it is essential to search for biological markers that can predict the patient’s prognosis.

Tumor Node Metastasis (TNM) stage was freauently used to predict the prognosis of ESCC patients in clinical (4). However, about 10% to 20% of ESCC patients have understaging after surgery, and its biological behavior is often more invasive than clinical staging (5). Similar to other tumors, esophageal cancer will form abundant tumor neovascularization during the occurrence and development. It is through the circulatory system that tumor cells will be transported to other parts of the body when they break through the neovascularization and enter the blood or lymphatic circulation.Therefore, vascular and lymphatic metastasis are the main modes of recurrence of esophageal cancer. Lymphovascular invasion (LVI) and lymph node metastasis are important factors affecting the prognosis of ESCC patients (6, 7). Clinically, there are often cases of negative lymph node metastasis but positive LVI, which suggests that LVI may be one of the pre-process or important steps of lymph node metastasis. LVI is one of the steps of tumor invasion and metastasis in esophageal cancer (8, 9). As a prognostic factor, its appearance indicates poor prognosis of patients, and has attracted more and more attention in recent years (10–12). In patients with LVI, the risk of recurrence is high, they need preoperative adjuvant treatment and intensive monitoring. For this reason, early identification of patients with high recurrence risk, especially those with early recurrence, is crucial for developing an individualized treatment plan for ESCC (13).

Currently, pathological examinations remain the gold standard for diagnosing esophageal cancer, and the evaluation of clinical stage before treatment mainly depends on the results of imaging examination. Accurate clinical staging determines the precise treatment of esophageal cancer (14, 15). The main imaging methods for the evaluation of esophageal cancer is based on computed tomography (CT), as a non-invasive imaging method, CT examination is used for the clinical TNM staging of esophageal cancer. Compared with plain CT, contrast enhanced CT(CECT) can not only reflect the morphological characteristics of the tumor, but also reflect the hemodynamic information of the tumor (16). Several studies have suggested that preoperative CT can be used to predict lymphovascular invasion of gastric and rectal cancer (14, 17–19). However, due to the low soft tissue resolution of CT, it is difficult to display the stratification of esophagus and the depth of invasion of tumor tissue, especially the early small lesions. For tumor heterogeneity, routine CT examination cannot provide more valuable information for clinical practice.

In recent years, radiomics has developed rapidly in tumor research (20). The research of radiomics in esophageal cancer includes the prediction of tumor staging, pathological characteristics, efficacy evaluation and prognosis (21, 22). Unlike traditional imaging, the application of imaging histological features can not only improve the accuracy of diagnosis, but also provide information that traditional imaging cannot provide. Therefore, radiomics has a very broad application prospect in the evaluation of esophageal cancer, and it is crucial for determining and adjusting a patient’s individualized treatment plan. It has been shown that radiomic features can be used to predict tumor grade, staging, treatment response, and survival for gastrointestinal cancer patients (23–25). In terms of predicting tumor LVI, radiomics has been successfully used to predict the LVI status of malignant tumors such as such as lung adenocarcinoma (26), gastric cancer (15), and rectal cancer (27). It has been shown by Li et al. that the radiomics model based on CECT can be used to predict LVI in ESCC (28). However, the value of radiomics in preoperative prediction of LVI status of esophageal cancer still needs to be further studied in large samples. Therefore, our study aimed to develop a nomogram model based on radiomic features that would predict LVI status in patients with ESCC before surgery, which could provide more information of incremental value for individualized treatment.

## 2 Materials and methods

### 2.1 Patient eligibility

326 esophageal cancer patients with radical esophagectomy and confirmed by postoperative pathology between June 2017 and December 2021 were analyzed retrospectively in our hospital. Inclusion criteria: (a) patients with radical resection of tumor and were confirmed as ESCC by postoperative pathology; (b) complete clinical datas; (c) Chest enhanced CT scan was performed within 7 days before surgery; (d) intact in paraffin, feasible pathological sections for hematoxylin-eosin (HE) staining and immunohistochemical (IHC) analysis; Exclusion criteria: (a) absence of complete pathological data or unclear LVI status; (b) preoperative local or systemic anti-tumor therapy; (c) poor image quality or obvious artifacts affect image evaluation (d) no identifiable lesion on CT images. According to the inclusion and exclusion criteria, 258 patients were included in the study.A training cohort(n=181) and a validation cohort(n=77) at a ratio of 7:3 were divided randomly.

### 2.2 Pathological evaluation

Two experienced pathologists used the 8th edition of the AJCC staging system to stage and classify the degree of differentiation (29), and determine the LVI status of the patients. The LVI was identified as tumor cell emboli within the space of the endothelial lining on HE-stained sections.

### 2.3 CT Image acquisition and analysis

A dual-source CT scanner (Siemens Somatom Definition, Munich, Germany) was used to scan the patients, and during a single breath-hold with the patient lying supine, the entire esophagus region is scanned. A 120kVp; 130mAS imaging acquisition system was used. Rotation time was 0.5 seconds. Colliding width was 64mm, pitch was 1.5:1. The field of view (FOV) was 350mm x 350mm; matrix was 512x512; 5mm layer thickness;5mm layer spacing. An enhanced CT scan was performed at 25-30 seconds following the injection of 1.5ml/kg of iohexol or ioverol contrast medium(Henrui Medicine, Lianyungang, China) into the ulnar vein at 2.5-3.0 ml/s with a high-pressure syringe.

The preoperative CT images were retrospectively analyzed and evaluated by picture archiving and communication system (PACS), and the optimal window width and window position were adjusted for the CT images of each patient. Image analysis was performed blinded by two experienced radiologists (8 and 15 years of CT reading experience, respectively),with no knowledge of clinical, pathological, and LVI status data. The CT images of each patient were read independently by an radiologist 1 (8 years) and reconfirmed by another senior radiologist 2 (15 years), and agreed upon in case of disagreement. Thickness of the normally dilated esophageal wall is about 3mm, while the patient with esophageal cancer shows local thickening or mass-like significant strengthening of esophageal wall, and the local thickening of the esophageal wall ≥5mm is abnormal thickening (30).

CT image features are as follows: (a) tumor location (b) tumor size: The tumor length and thickness were measured using CECT; (c) tumor-to-normal wall enhancement ratio (TNR): The ratio of the mean CT value of the tumor to the normal esophageal wall is calculated;(d) According to Griffin et al., clinical T staging refers to their criteria for evaluating cancer patients (30). Depending on the number of metastatic lymph nodes in different regions, the clinical N stage is determined, Moreover, the assessment of metastatic lymph nodes is based on the shortest diameter plus the node axis ratio of the enlarged lymph nodes (31, 32). The clinical AJCC stage(cAJCC stage) were according to the eighth edition of the AJCC staging system (29).

### 2.4 Tumor segmentation

The CT images of all patients were uploaded to the open-source software “ITK-SNAP” (www.itksnap.org). The region of interest (ROI) was manually delineated by two radiologists with more than five years of experience along the tumor edge to achieve tumor segmentation. Because tumors are heterogeneous, the three-dimensional (3D) ROI should encompass the entire lesion. After delineation is complete, modify the ROI with reference to the MPR image.

### 2.5 Radiomics feature extraction

The image preprocessing and radiomic feature extraction were performed by PyRadiomics 2.1.2 software package. 1316 radiomics features were extracted with 18 first-order features, 554 texture features and 744 wavelet features. All characteristic parameters are standardized by Z-score according to the training set data. For the purpose of exploring radiomics features’ intra-observer stability,radiologist 1 repeated the independent segmentation and feature extraction of 30 randomly selected patients within 1 week; to explore the inter-observer stability of radiomics features, radiologist 2 performed ROI independent segmentation and feature extraction on 30 randomly selected patients. The reproducibility of feature extraction was assessed using intra- and inter-class correlation coefficients (ICCs).

### 2.6 Radiomics feature selection and model construction

**Figure 1:**
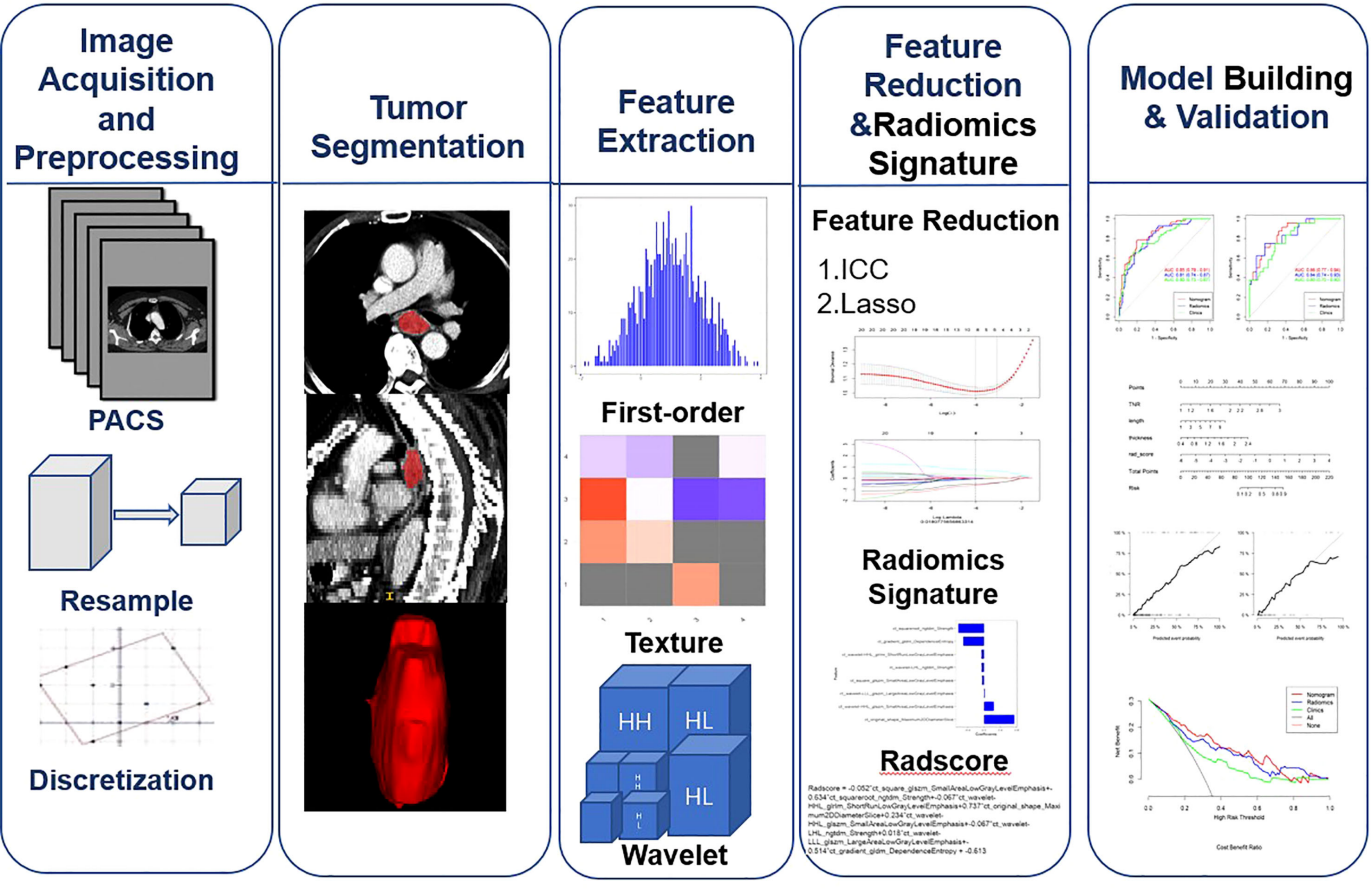
Workflow for image preprocessing,image segmentation, radiomics feature extraction, feature reduction, and model building and validation for this study.

This study aggregates multiple algorithms for dimensionality reduction of high-dimensional data:(1)Feature parameters that are highly stable (ICCs> 0.90) in both intra- and inter-observer consistency tests were selected for analysis.(2) The top 20 radiomics feature parameters ranked by feature score or importance in the combined maximal relevance and minimal redundancy (MRMR). (3) Further screening of the feature parameters was performed using the least absolute shrinkage and selection operator (LASSO),an optimal weight parameter was selected by 10-fold cross-validation, and a linear combination formula was computed to generate the radiomics signature.The radiomics workflow is presented in **Figure 1**.

### 2.7 Statistical analysis

R software version 3.6.3 (Auckland, New Zealand) and SPSS version 22.0 for Windows (Chicago, USA) was performed to statistical analysis. In clinical and pathological analysis, continuous variables are reported as means + standard deviations and categorical variables as counts (%).The chi-square test was used for categorical variables. The continuous variables were analyzed by independent sample t test if they conformed to normal distribution, otherwise Mann-Whitney U test were used. The receiver operating characteristic (ROC) curve was analyzed for each model. Then, the area under the curve (AUC), the accuracy, the sensitivity, the specificity, the positive predictive value(PPV), and the negative predictive value(NPV) were calculated. Comparing prediction models using AUC values was done using the Delong test. The goodness of fit of the model was determined by drawing calibration curves. In order to calculate the clinical effectiveness, a decision curve analysis (DCA) was performed.The statistical significance levels were all two-sided, and the p-value <0.05 was considered to indicate statistical significance.

## 3 Results

### 3.1 Patient characteristics

**Table 1** shows the characteristics of patients in the training and validation cohorts. 181 patients were collected in the training cohort and 77 patients in the validation cohort. In the training cohort, no significant differences were found in age, gender and tumor location between LVI(-) and LVI(+) (p>0.05). However, significant differences were found in length, thickness, TNR, cT stage, cN stage, and cAJCC stage between LVI(-) and LVI(+) (p<0.05). In the validation cohort, no significant differences were found in age, gender, tumor location, TNR and cN stage between LVI(-) and LVI(+) (p>0.05). While significant differences were found in length, thickness, cT stage, and cAJCC stage between LVI(-) and LVI(+) (p<0.05).

**Table 1:** Characteristics of Patients in the Training and Validation Cohorts.

Characteristic	Training cohort (n=181)	Validation cohort (n=77)	P value	Training cohort (n=181)		P value	Validation cohort (N=77)		P value
				LVI(-) (n=125)	LVI(+) (n=56)		LVI(-) (n=53)	LVI(+) (n=24)	
Age	66.29 ± 7.35	66.44 ± 6.83	0.875	66.62 ± 6.83	65.54 ± 8.41	0.1336	66.73 ± 5.35	65.79 ± 9.42	0.650
Gender			0.923			0.847			0.566
Male	121(66.9%)	51(66.2%)		83(45.86%)	38(20.99%)		34(44.16%)	17(22.08%)	
Female	60(33.1)	26(33.8%)		42(23.20%)	18(9.94%)		19(24.68%)	7(9.09%)	
location			0.436			0.871			0.584
Up	29(16.0)	10(13.0%)		20(11.05%)	9(4.97%)		8(10.39%)	2(2.60%)	
Medium	76(42.0)	28(36.4%)		54(29.83%)	22(12.15%)		20(25.97%)	8 (10.39%)	
Low	76(42.0)	39(50.6%)		51(28.18%)	25(13.81%)		25(32.47%)	14(18.18%)	
Length	3.79 ± 1.50	3.82 ± 1.72	0.895	3.45 ± 1.31	4.54 ± 1.63	<0.001	3.42 ± 1.38	4.69 ± 2.09	0.002
Thickness	1.31 ± 0.38	1.30 ± 0.38	0.921	1.20 ± 0.33	1.55 ± 0.36	<0.001	1.17 ± 0.30	1.59 ± 0.36	<0.001
TNR	1.54 ± 0.30	1.49 ± 0.29	0.274	1.49 ± 0.28	1.63 ± 0.33	0.004	1.18 ± 0.28	1.52 ± 0.34	0.036
cT			0.660			<0.001			<0.001
T1	9(5.0%)	5(6.5%)		9(4.97%)	0		5(6.49%)	0	
T2	8(48.6%)	40(51.9%)		78(43.09%)	10(5.52%)		37(48.05%)	3(3.90%)	
T3	52(28.7%)	23(29.9%)		30(16.57%)	22(12.15%)		10(12.99%)	13(16.88%)	
T4	32(17.7%)	9(11.7%)		8(4.42%)	24(13.26%)		1(1.30%)	8(10.39%)	
cN			0.871			0.013			<0.001
N0	113(62.4%)	50(64.9%)		87(48.07%)	26(14.36%)		39(50.65%)	11(14.29%)	
N1	50(27.6%)	19(24.7%)		26(14.36%)	24(13.26%)		14(18.18%)	5(6.49%)	
N2	17(9.4%)	8(10.4%)		12(9.6%)	6(3.31%)		0	8(10.39%)	
N3	1(0.6%)	0(0%)							
cAJCC			0.426			<0.001			<0.001
I	9(4.9%)	5(6.5%)		9(4.97%)	0		5(6.49%)	0	
II	106(58.6%)	52(67.5%)		89(49.17%)	17(9.39%)		44(57.14%)	8(10.39%)	
III	34(18.8%)	11(14.3%)		19(10.50%)	15(8.29%)		3(3.90%)	8(10.39%)	
IV	32(17.7%)	9(11.7%)		8(4.42%)	24(13.26%)		1(1.30%)	8(10.39%)	

### 3.2 Feature selection and radiomics signature construction

**Figure 2:**
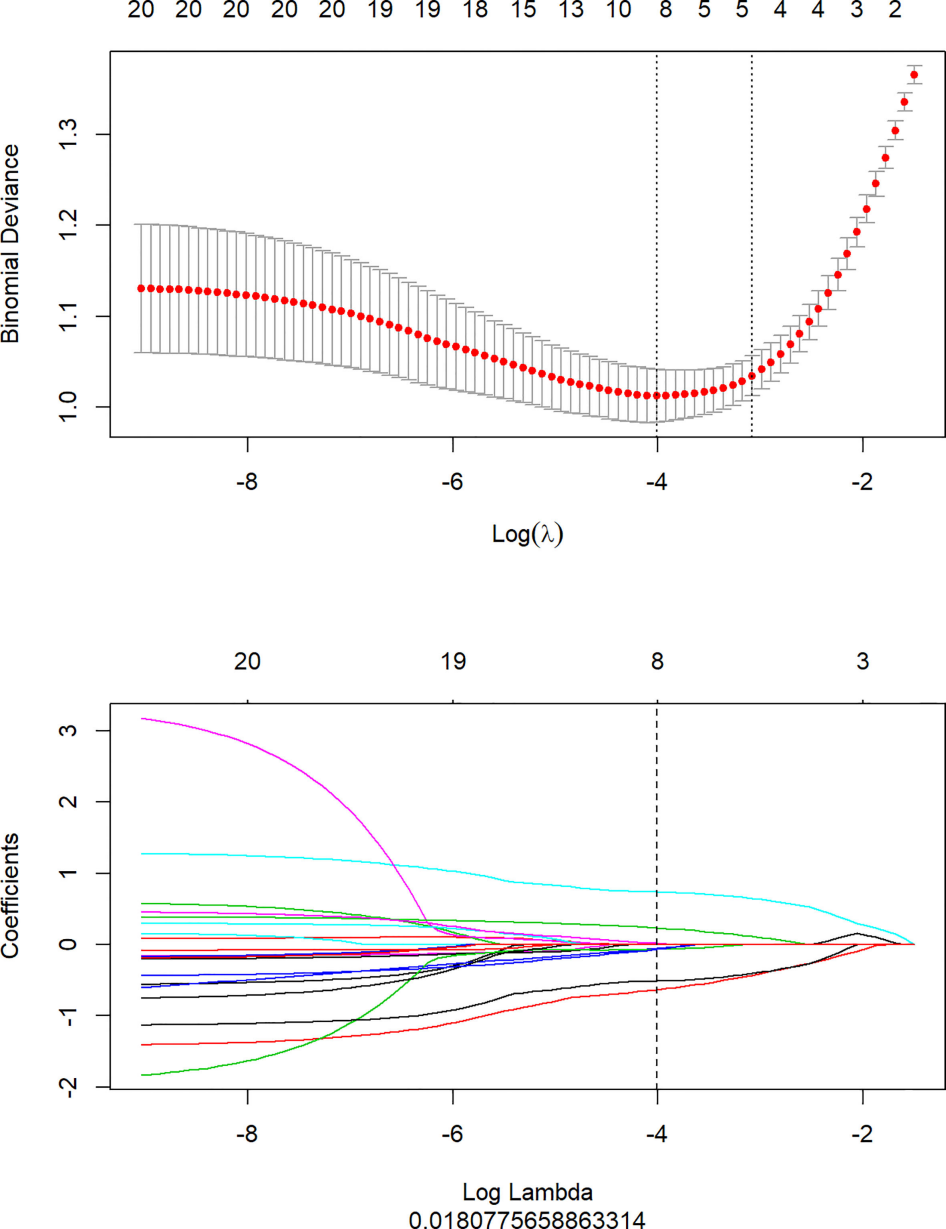
Texture feature selection using the least absolute shrinkage and selection operator (LASSO) binary logistic regression model.

**Figure 3:**
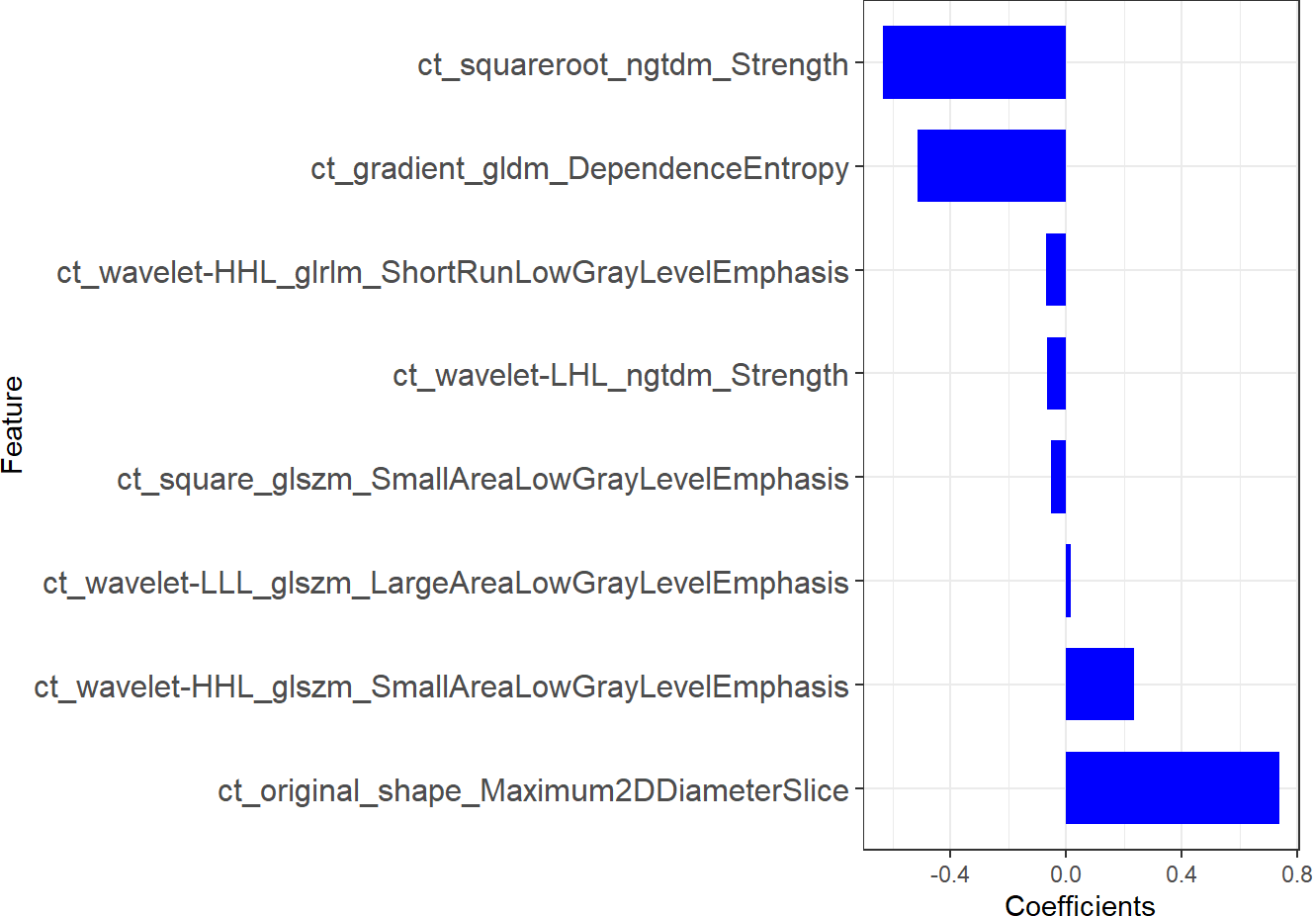
The most predictive subset of radiomics features for predicting LVI in ESCC.

1316 features were extracted from segmented pretreatment CT images based on 258 patients with ESCC. 978 features were preserved for further analysis, after the reproducibility test by using an ICCs> 0.90. To help further reduce the number of features while retaining the most relevant and informative ones, we chose the top 20 features based on their MRMR score. By selecting the top 20 features with high MRMR scores, we ensure that these features are both relevant and non-redundant, and thus less likely to be removed by Lasso. This approach can lead to a more efficient and accurate model with improved interpretability. Then, we removed features with Pearson correlation coefficients greater than 0.90 to eliminate highly correlated features, while retaining sufficient features for LASSO model selection. The LASSO was used to further reduce the dimension (**Figure 2**). Lasso dimensionality reduction is a technique that shrinks the coefficients of less important features to zero, effectively removing them from the model. After the determined number of features, the feature subset with the strongest predictive power was selected and the corresponding coefficients were evaluated. Finally, we screened out 8 important radiomics features and constructed radiomics signature (**Figure 3**). Rad-score was calculated by summing the selected features weighted by their coefficients. AUC of the radiomics signature performance was 0.805 in the training cohort (95% CI: 0.740-0.860), and 0.836 in the validation cohort (95% CI: 0.735-0.911).

### 3.3 Clinical model development and validation

An univariate analysis of clinical imaging features revealed that TNR, tumor length, thickness, and cN stage were significantly related to LVI.Multivariate analysis showed that TNR, tumor length and thickness were independent predictors of LVI. (**Table 2**). We ultimately used logistic regression to construct a clinical model, including factors such as TNR, tumor length and thickness. The results showed that the AUC was 0.803 in the training cohort and 0.826 in the validation cohort.

**Table 2:** Univariate and Multivariate analysis to identify significant factors for LVI.

	Univariate	P	Multivariate	P
	OR (95% CI)		OR (95% Cl)	
Age	0.99(0.95-1.02)	0.280	—	—
Gender		0.634*	—	—
Male	Reference		—	—
Female	0.87(0.50-1.53)	0.634	—	—
Tumor location		0.663*	—	—
Up	Reference		—	—
Medium	1.03(0.46-2.33)	0.940	—	—
Low	1.31(0.59-2.90)	0.511	—	—
Length	1.65(1.35-2.01)	<0.001	1.36(1.07-1.72)	0.011
Thickness	23.62(9.35-59.68)	<0.001	16.32(5.89-45.17)	<0.001
TNR	3.20(1.33-7.66)	0.009	4.87(1.58-15.02)	0.006
cT stage	NA	NA	—	—
cNstage		0.001*		0.117
N0	Reference		Reference	
N1	2.47(1.35-4.51)	0.003	2.20(1.09-4.43)	0.027
N2	4.33(1.82-10.35)	0.001	2.20(0.79-6.15)	0.133
cAJCC	NA	NA	—	—

*Overall P value. NA, Not Applicable.

### 3.4 Radiomics nomogram development and validation

**Figure 4:**
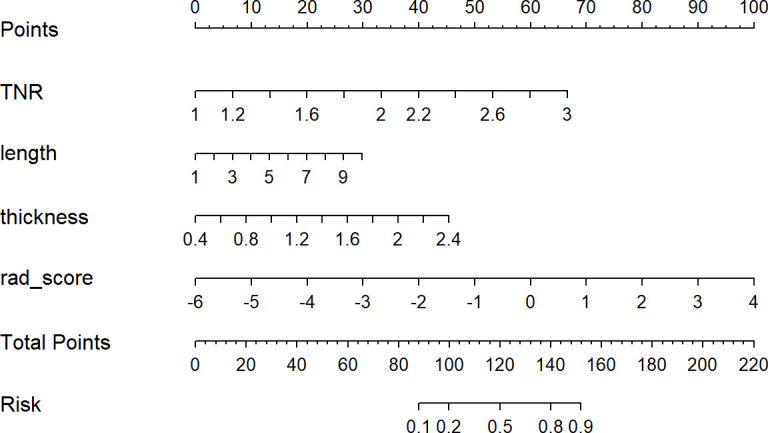
Developed radiomics nomogram. The radiomics nomogram was developed in the primary cohort, with the rad-score, TNR, length, and thickness incorporated.

**Figure 5:**
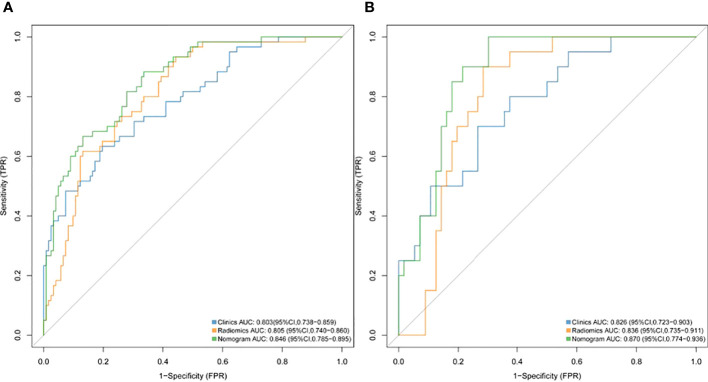
ROC curves of the radiomics, clinical and nomogram models for predicting LVI in the training cohort **(A)** and validation cohort **(B)**.

**Figure 6:**
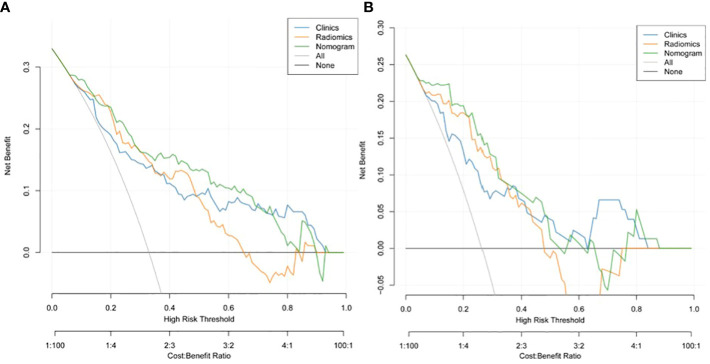
Decision curve analysis (DCA) of the training cohort **(A)** and validation cohort **(B)**. DCA indicated that using the nomogram model to predict LVI would be more beneficial than a "treat-all" or "treat-none" regimen.

A nomogram model was developed using multivariate logistic regression and includes TNR, tumor length, thickness, and radiomics signature (**Figure 4**). A good calibration performance of the nomogram calibration curve was showed in the training and validation cohorts, and no statistically significant difference was found between the training and validation cohorts in the Hosmer-Lemeshow test (P>0.05), indicating no deviation from the fit. The accuracy of the nomogram for LVI prediction in the training and validation cohorts was 79.3% and 76.8%, respectively.The sensitivity was 78.6% and 91.7%, and the specificity was 80.0% and 61.8%, respectively. The AUC of the nomogram for LVI prediction in the training and validation cohorts was 0.846(0.785-0.895) and 0.870(0.774-0.936), respectively (**Table 3**), (**Figure 5**). The result of the Delong test showed that in the training set, the Nomogram model performed better than the Clinical model and the Radiomics model with significant difference (Z=2.239 and 1.825, p=0.025 and 0.048, respectively), while there was no significant difference in diagnostic performance between the Clinical model and the Radiomics model (Z=0.052, p=0.958). In the validation set, the Nomogram model performed better than the Clinical model with significant difference (Z=1.310, p=0.039), while there was no significant difference in diagnostic performance between the Nomogram model and the Radiomics model (Z=1.116, p=0.190). To sum up, Nomogram model have better diagnostic performance compared with Clinical model. DCA showed that in the training set, the Nomogram model generated higher net benefits compared to the clinical model and the imaging omics model within the probability range of 0 to 0.670. In the test set, the probability range was 0 to 0.462, and the Nomogram model still generated higher net benefits than the other models (**Figure 6**).

**Table 3:** Diagnostic performance of different prediction models.

Model	AUC (95%CI)	Accuracy (%)	Sensitivity (%)	Specificity (%)	PPV (%)	NPV (%)
Training cohort(n=181)						
Clinics	0.803(0.738-0.859)	75.5	66.1	84.8	66.1	84.8
Radiomics	0.805(0.740-0.860)	74.9	92.9	56.8	49.1	94.6
Nomogram	0.846(0.785-0.895)	79.3	78.6	80.0	63.8	89.3
Validation cohort(n=77)						
Clinics	0.826 (0.723-0.903)	74.3	95.8	52.8	67.0	92.6
Radiomics	0.836 (0.735-0.911)	77.3	62.5	92.1	88.8	71.1
Nomogram	0.870 (0.774-0.936)	76.8	91.7	61.8	70.6	88.2

## 4 Discussion

Researchers have found that LVI is an independent predictor of survival in patients with ESCC (33, 34). According to AJCC/UICC guidelines, LVI is not yet included as an independent prognostic indicator for esophageal cancer in the TNM staging system. The accurate preoperative prediction of LVI status, however, is crucial for patients to develop aggressive treatment plans tailored to their individual needs. Clinically, more aggressive treatment is required for patients suspected of tumor microvascular invasion.The scope of surgery can be expanded or preoperative adjuvant therapy can be administered.In the present study, a diagnostic nomogram for preoperative prediction of LVI were developed and validated in ESCC patients. The nomogram included four items (radiomics signature, tumor length, thickness, and TNR). Radiomics and image features derived from CECT may be used to formulate a nomogram to predict ESCC LVI preoperatively. Patients with ESCC will benefit from this novel approach by providing risk stratification and decision support.

To construct the radiomics signature, the LASSO method was used to narrow the regression coefficients to test the association of the prediction results. As a result of using the univariate association method for selecting predictors, our approach performs better than that of using the multivariate association method. Additionally, it creates a radiomics signature which combines selected features (35). In this study, important radiomics features were screened out from 978 candidate radiomics features, and 8 radiomics features that could predict the LVI were finally selected, among which wavelet filter contributed the most information (n =4). This is followed by squares (n=1), square roots (n=1), gradients (n=1), and original features (n=1). These findings suggest that the wavelet filter contains the most tumor heterogeneity information and is the best available radiomics feature (one in two), consistent with the results of other CT-based radiomics studies. The wavelet feature reflects the multi-frequency information of multiple dimensions of tumor. The square reflects the square of the image intensity; The square root reflects the square root of the image intensity; A gradient reflects a change in the gradient of the voxel in the image. Our study used these four filters to quantify tumor heterogeneity. Maximum2Ddiameter is of great value in the original shape feature. Maximum2Ddiameter is of great value in the selected original shape feature.It is the maximum paired euclidean distance between the vertices of the mesh on the tumor surface. Gray Level Size Zone (GLSZM) quantifies gray areas in an image, which are defined as the number of adjacent voxels with the same gray intensity. In this study GLSZM significant characteristics is Small Area Low Gray Level Emphasis (SALGLE) and Large Area Low Gray Level Emphasis (LALGLE). SALGLE measures the grey value lower area of the small size of the ratio of joint distribution in the image. LALGLE measures the proportion of the joint distribution of large size areas with low gray value in the image. Neighbouring Gray Tone Difference Matrix(NGTDM)represents the difference between the gray value in one area and the average gray value in an adjacent area. A meaningful subcharacteristic of NGTDM is strength, which reflects the measure of the original element in the image. When the intensity of the image changes slowly, but the coarse difference of the intensity of the gray level is large, the value is higher. Gray Level Run Length Matrix(GLRLM)quantifies grayscale run, defined as the length of the number of consecutive pixels with the same grayscale value. GLRLM features assess the percentage of pixels/voxels within ROI, which reflects “graininess”. GLRLM meaningful characteristics is Short Run Low Gray Level Emphasis (SRLGLE), it quantitatively reflect has lower grey value of shorter run lengths of joint distribution. Gray Level Dependence Matrix(GLDM)quantifies the dependence of image gray scale. A significant subfeature of GLDM is dependence entropy, and the characteristic of heterogeneous enhancement accurately reflects the grayscale heterogeneity of entropy. Our results show that among the sub-features: the larger the values of SALGLE(wavelet), LALGLE(wavelet) and Maximum2Ddiameter(primitive), the greater the tumor heterogeneity and the greater the risk of LVI in ESCC.; the smaller the values of SRLGLE(wavelet), strength (wavelet, square root), SALGLE(square) and DependenceEntropy(gradient), the greater the tumor heterogeneity and the greater the risk of LVI.Finally, we use these 8 radiomics features to construct radiomics signature.This signature has high stability and low redundancy, and retains the features of correlation and stability with LVI. Based on this radiomics signature, we established a radiomics prediction model. Radiomics model showed good diagnostic performance in the training cohort and validation cohort, with AUCs of 0.806 and 0.836, respectively. Accordingly, the model predicts with high accuracy and stability, which is consistent with Li et al’s findings (28). So, the model is expected to provide a reliable reference for clinical decision-making.

Multivariate analysis revealed that TNR was an independent predictor of LVI in our clinical model. Similarly to what we found, Komori et al. found that TNR is associated with vasolymphatic invasion of tumors (19). It is believed that the VEGF family is actively involved in neovascularization and lymphangiogenesis of tumors (31) and that a close association exists between neovascularization in tumors and LVI in esophageal cancer (36, 37). LVI may therefore be visible in arterial phase images revealing changes in vascular morphology and hemodynamics (38). The results of our study could be explained theoretically by this. In addition, tumor length and thickness are independent predictors of LVI.The maximum length and thickness of the tumor reflect the extent and depth of tumor invasion and LVI is linked to these factors. With increasing tumor invasion, LVI incidence increased. On CECT images, the identification of tumor areas often depends on the effect of esophageal wall thickness on the degree of invasion. The CECT shows certain advantages in measuring tumor length and thickness, and can be used for preoperative T staging (16). There was an independent correlation between tumor length and thickness and LVI in our study. Accordingly, tumor length and thickness can more accurately reflect tumor invasion than clinical T staging, and thus LVI status can be better predicted.

Given that our constructed clinical model found that multiple CECT-based image features were shown to be significantly associated with LVI status. A nomogram model combining CECT radiomics features and image features was developed to further improve the predictive power.According to the results, the AUC in the training cohort was 0.846,the accuracy was 79.3%, the sensitivity was 78.6%, the specificity was 80.0%, the positive predictive value was 63.8%, and the negative predictive value was 89.3%; the AUC in the validation cohort was 0.870, and the accuracy was 76.8%, the sensitivity is 91.7%, the specificity is 61.8%, the positive predictive value is 70.6%, and the negative predictive value is 88.2%. It can be seen that this nomogram has good predictive performance, and the predicted value of the nomogram is verified by the calibration curve. There was good agreement with pathological results. In a previous study, Chen et al. (15) developed a model for preoperative prediction of LVI status of gastric cancer based on CECT radiomics features in arterial and venous phases. As a result of the combined arterial-venous radiomics features along with clinical risk factors, the AUC of the combined model was 0.8565 in the training cohort and 0.7929 in the validation cohort,the diagnostic performance of which was good. Compared to our study, this combined model performed similarly in diagnostics. Clinically, venous phase CECT is not routinely used for esophageal cancer, and arterial phase CECT is more frequently used.In our study, we used arterial phase CECT images instead of previous studies to create a radiomics model. Researchers developed a radiomics model to evaluate LVI status in rectal cancer using multimodal MRI and venous-phase enhanced CT images (27), and comparing the combined model to other models, it showed the best diagnostic performance.Incorporating multimodal MR into our radiomics model may improve predictive accuracy.However, MRI has not yet been used as a routine preoperative examination for esophageal cancer, and most ESCC patients undergo routine CECT scans before surgery. We can make full use of these imaging data to predict the occurrence of LVI, which is also an economical method. The CECT-based radiomics nomogram model we developed and validated is capable of generating individual probabilities for predicting LVI by integrating readily available preoperative radiomics and image features. Preoperative individualized prediction of LVI risk using an easy-to-use scoring system, which is in line with the current trend of personalized medicine.

Last but not least, a nomogram is necessary to explain an individual’s need for additional treatment. Performance, identification, and calibration of risk prediction do not account for the clinical consequences of this degree of accuracy or miscalibration (39–41). Thus, we evaluated whether using radiomics nomograms would improve patient outcomes to demonstrate clinical utility.To achieve this goal, the present study employed decision curve analysis rather than a multi-institutional prospective validation of nomograms. Due to the heterogeneity of clinical data and CT image parameters among different institutions, the nomogram is largely inconsistent with clinical practice. Based on threshold probability-based observations of clinical outcomes, net benefits can be calculated (42). According to decision curves, using radiomics nomograms to predict LVI in the current study provided more benefit than treating everyone or not treating anyone if the threshold probability of patient or physician was 10%.

However, our study has some limitations. First, there may have been some selection bias in this study because it was retrospective and included only patients with esophageal cancer who had undergone surgery. Second, this study is a single-center study that only included cases from one center and lacked external validation. There are certain differences in CT equipment parameter settings and imaging algorithms in different hospitals, which may result in poor stability of omics characteristics. Therefore, the model still has certain limitations, and further multi-center validation is needed to obtain more convincing evidence with a larger sample size. Third, this study only includes radiomics, and lacks the integration of multiple omics such as genomics and proteomics, so there are natural limitations. Finally, the prognostic value of CT radiomics features in ESCC patients with LVI was not further investigated in this study, which may be our next research work.

## 5 Conclusion

In general, in this study, we established a non-invasive, cost-effective, and individualized LVI prediction model based on preoperative CECT images. The model includes radiomics features and image features, and has good accuracy in predicting LVI with ESCC. Large-scale multicenter retrospective validation and prospective randomized clinical trials await further validation.

## Data availability statement

The datasets presented in this article are not readily available because they are privately owned by the affiliated Huaian No.1 People’s Hospital of Nanjing Medical University and are not made public. Requests to access these datasets should be directed to the corresponding author WC, wchen74@163.com.

## Ethics statement

The studies involving human participants were reviewed and approved by Institutional review board of the Affiliated Huaian No.1 People’s Hospital of Nanjing Medical University(KY-2022-045-01. Written informed consent for participation was not required for this study in accordance with the national legislation and the institutional requirements.

## Author contributions

Guarantor of integrity of the entire study: WC. Study concepts and design: YW. Literature research: GB. Clinical studies: WH. Manuscript preparation: HZ. All authors contributed to the article and approved the submitted version.
